# Influence of Temperature on Free Radical Generation in Propolis-Containing Ointments

**DOI:** 10.1155/2016/7292379

**Published:** 2016-08-03

**Authors:** Paweł Olczyk, Pawel Ramos, Katarzyna Komosinska-Vassev, Lukasz Mencner, Krystyna Olczyk, Barbara Pilawa

**Affiliations:** ^1^Department of Community Pharmacy, School of Pharmacy and Division of Laboratory Medicine in Sosnowiec, Medical University of Silesia, Kasztanowa 3, 41-200 Sosnowiec, Poland; ^2^Department of Biophysics, School of Pharmacy and Division of Laboratory Medicine in Sosnowiec, Medical University of Silesia, Jednosci 8, 41-200 Sosnowiec, Poland; ^3^Department of Clinical Chemistry and Laboratory Diagnostics, School of Pharmacy and Division of Laboratory Medicine in Sosnowiec, Medical University of Silesia, Jednosci 8, 41-200 Sosnowiec, Poland

## Abstract

Free radicals thermally generated in the ointments containing propolis were studied by electron paramagnetic resonance (EPR) spectroscopy. The influence of temperature on the free radical concentration in the propolis ointments was examined. Two ointment samples with different contents of propolis (5 and 7%, resp.) heated at temperatures of 30°C, 40°C, 50°C, and 60°C, for 30 min., were tested. Homogeneously broadened EPR lines and fast spin-lattice interactions characterized all the tested samples. Free radicals concentrations in the propolis samples ranged from 10^18^ to 10^20^ spin/g and were found to grow in both propolis-containing ointments along with the increasing heating temperature. Free radical concentrations in the ointments containing 5% and 7% of propolis, respectively, heated at temperatures of 30°C, 40°C, and 50°C were only slightly different. Thermal treatment at the temperature of 60°C resulted in a considerably higher free radical formation in the sample containing 7% of propolis when related to the sample with 5% of that compound. The EPR examination indicated that the propolis ointments should not be stored at temperatures of 40°C, 50°C, and 60°C. Low free radical formation at the lowest tested temperatures pointed out that both examined propolis ointments may be safely stored up to the temperature of 30°C.

## 1. Introduction

Propolis is a natural agent produced by* Apis mellifera*, resulting from the addition of salivary enzymes to resins collected from various plant sources, mixed with wax, and used for narrowing nest hive entrances, sealing gaps, and embalming dead organisms inside the hive, thus preventing decomposition and spreading of odors [1–3].

Although the chemical composition and biological activity of propolis are highly changeable due to the variability of plant species occurring around the hive, from which the bees collect the exudates, the mentioned apitherapeutic comprises approximately 50% plant resins, 30% waxes, 10% essential and aromatic oils, 5% pollens, and 5% of impurities [2, 4, 5]. Until the present time, over 300 ingredients, contained in the biologically potent fractions such as flavonoids, phenolics, and aromatic compounds, have been identified in propolis [4, 5]. The mentioned constituents determine the properties of propolis including anti-inflammatory, antimicrobial, antioxidant, antitumor, antiulcer, regenerating, and anti-HIV activities [3, 4, 6–10].

Due to special properties, high activity, and a broad application of propolis-containing ointments in medicine and pharmacy [1–5], they should not contain large amounts of free radicals. The ointments should be stored at specified conditions, among others at a proper temperature, which is safe for the chemical structure of the therapeutic constituents of the ointment. Thermal formation of free radicals should not occur. Too high temperature may lead to undesirable free radicals thermal formation in the ointment environment. The rupturing of the chemical bonds of the ointment compounds, accompanied by the generation of unpaired electrons, may weaken the therapeutic efficacy of the mentioned medicine. The toxic impact of free radical reactions on the exposed skin must be avoided. The aim of this work was to determine the concentrations and properties of thermally formed free radicals in two ointments different in terms of the amounts of propolis. Moreover, different temperatures of the propolis ointment storage were analyzed.

The performed EPR analysis had the innovatory character since the free radical scavenging activity of 5% and 7% ointment samples was not examined by electron paramagnetic resonance earlier probably because of technical and analytical difficulties. In our work we implemented for the first time electron paramagnetic resonance (EPR) spectroscopy as an experimental tool for the examination of broadened EPR lines and fast spin-lattice interactions present in the used propolis formulations. Moreover, the instrumental method applied by us was nondestructive and demanded only a small amount of sample for the experimental estimation.

The undertaken study is supposed to broaden our earlier knowledge about the usefulness of electron paramagnetic resonance spectroscopy studies in the assessment of propolis antioxidative properties [11–13].

## 2. Experimental

### 2.1. Samples Characterization

The subjects of the study were propolis-containing ointments at two different propolis concentrations, 5% and 7%, respectively. The abovementioned topical propolis ointments were obtained from the Apiary “Barć” Galenowa Wytwórnia Farmaceutyczna, Kamianna, Poland, and were authorized under the certificate RK/221957/2008 for 5% propolis ointment and RK/157056/2006 for the ointment containing 7% of propolis.

The applied propolis ointments at concentrations of 5% and 7% were chosen on the basis of the literature knowledge and experimental studies investigating the anti-inflammatory effect of the topical formulation containing propolis at abovementioned concentrations [14, 15]. On the other hand, 5% and 7% propolis ointments are commonly used as topically applied propolis extracts that belong to the natural product available on the polish pharmaceutical market, being also listed in the Journal of Medicinal Products List admitted to trade on Polish territory.

### 2.2. Thermal Treatment of the Ointment Samples

The ointments containing propolis were heated at temperatures of 30°C, 40°C, 50°C, and 60°C for 30 min. The mentioned temperatures of heating were chosen as the potential storage temperatures of the medicine. These temperatures reflected the possible thermal conditions in the storage environment. A professional hot air oven produced by Memmert Firm (Germany) with temperature programmer with additional air exchange rates controlled by AtmoControl software was used. The mentioned experimental device possesses extended temperature protection ensured by the integrated PT100 sensor for independent temperature monitoring.

### 2.3. EPR Measurements

Free radicals in thermally treated ointments were examined at room temperature 15 minutes after heating. The individual samples were located in thin-walled glass tubes of high paramagnetic purity with the external diameter of 1 mm. The empty glass tubes were free of EPR signals. The masses of the samples in the tubes were determined as the difference of the mass of the tube with the ointment and the mass of the empty tube by the use of Sartorius CPT weight (Germany). To measure the EPR spectra the samples in glass tubes were placed in the resonance cavity of the EPR spectrometer.

The measurements were done by an X-band (9.3 GHz) electron paramagnetic resonance spectrometer of Radiopan Firm (Poznań, Poland) with a magnetic modulation of 100 kHz and a system of numerical data acquisition, Rapid Scan Unit of Jagmar Firm (Cracow, Poland). The lines were detected as the first-derivative EPR spectra in the range of microwave power from 2.2 mW to 70 mW. The microwave frequency was measured by MCM101 recorder produced by EPRAD Firm (Poznań, Poland).

The following parameters of the EPR spectra were determined: *g*-factors [±0.0002], amplitudes (*A*) [±0.01 a.u.], integral intensities (*I*) [±0.02 a.u.], and linewidths (Δ*B*_pp_) [±0.02 mT]. The *g*-factors were calculated from the resonance condition as [16, 17]: *g* = *hν*/*μ*_*B*_ *B*_*r*_, where *h* is Planck constant, *ν* is microwave frequency, *μ*_*B*_ is Bohr magneton, and *B*_*r*_ is induction of resonance magnetic field. The influence of microwave power on amplitudes (*A*) and linewidths (Δ*B*_pp_) was examined. Integral intensities (*I*) were obtained by double integration of the first-derivative EPR curves. Integral intensities (*I*) were used in calculations of free radical concentrations (*N*) in the samples.

The references in free radical concentrations (*N*) studies were ultramarine [18, 19] and a ruby crystal (Al_2_O_3_:Cr^3+^). Free radical concentrations (*N*) in the ointments were calculated according to the following formula [9, 10]: *N* = *N*_*u*_[(*W*_*u*_ *A*_*u*_)/*I*_*u*_]·[*I*/(*WAm*)], where *N*_*u*_ is number of paramagnetic centers in the reference and ultramarine; *W*, *W*_*u*_ are receiver gains for the tested sample and ultramarine; *A*, *A*_*u*_ are amplitudes of ruby signal for the tested sample and the ultramarine; *I*, *I*_*u*_ are integral intensities for the tested samples and ultramarine, and *m* is mass of the sample. Ultramarine was obtained from Professor Andrzej B. Wieckowski from the Institute of Molecular Physics of Polish Academy of Sciences in Poznan (Poland) and the Institute of Physics of University in Zielona Góra (Poland).

The EPR measurements and analysis were performed by professional spectroscopic programs of Jagmar Firm (Cracow, Poland), LabView (National Instruments, USA), and ORIGIN 2016, OriginLab Corporation (Boston, USA).

## 3. Results and Discussion

**Figure 1:**
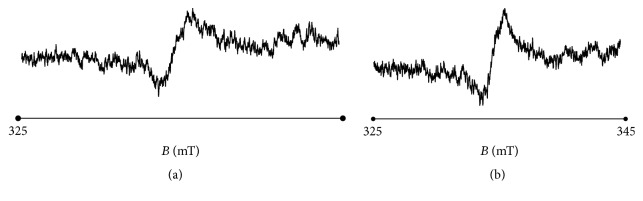
EPR spectra of the ointments containing (a) 5% and (b) 7% of propolis heated at the temperature of 60°C. *B*: magnetic induction. The spectra were measured with low microwave power of 2.2 mW.

The tested unheated propolis ointments did not contain free radicals. Free radicals were thermally formed in these samples. EPR signals were not observed for the unheated samples, which indicated the fact that they were diamagnetic and free of unpaired electrons. The propolis ointments become paramagnetic after thermal treatment at temperatures of 30°C, 40°C, 50°C, and 60°C. For the heated propolis samples, the EPR lines with free radical *g* factor of 2.00 were measured. The exemplary EPR spectra for the ointments containing 5% and 7% of propolis heated at the temperature of 60°C were shown in Figures 1(a) and 1(b), respectively.

**Figure 2:**
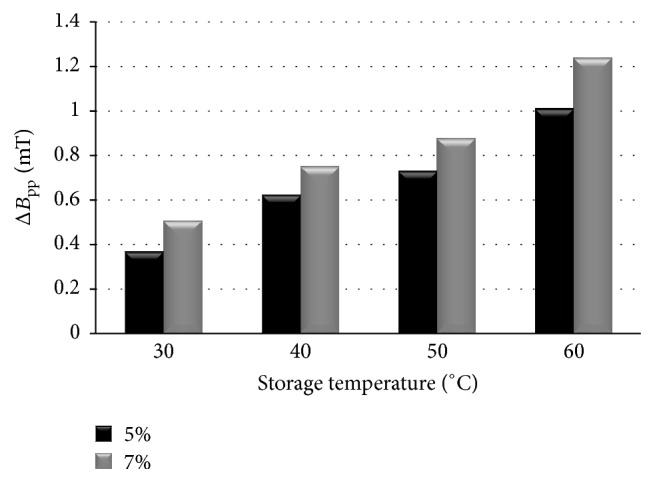
The influence of the heating temperature on linewidths (Δ*B*_pp_) of the EPR spectra of the ointments containing 5% and 7% of propolis. The data for the measurements with the microwave power of 2.2 mW.

**Figure 3:**
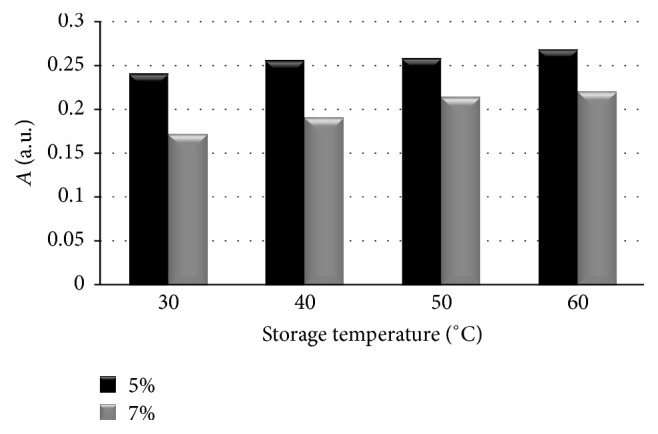
The influence of the heating temperature on amplitudes (*A*) of the EPR spectra of the ointments containing 5% and 7% of propolis. The data for the measurements with the microwave power of 2.2 mW.

**Figure 4:**
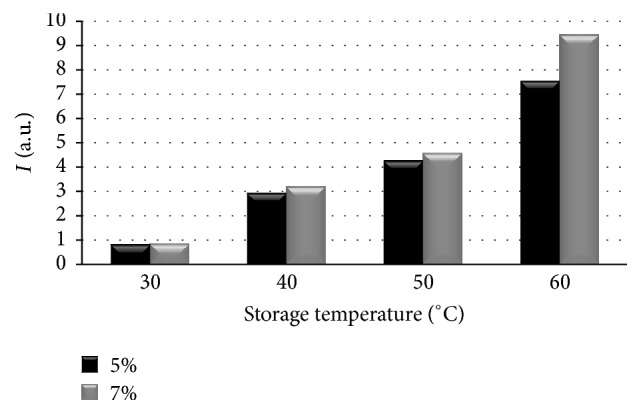
The influence of the heating temperature on integral intensities (*I*) of the EPR spectra of the ointments containing 5% and 7% of propolis. The data for the measurements with the microwave power of 2.2 mW.

The spectral parameters, linewidths (Δ*B*_pp_), amplitudes (*A*), and integral intensities (*I*), for the two ointments heated at different temperatures (30–60°C), were compared. The linewidths (Δ*B*_pp_) of the EPR lines of the ointments containing 5% and 7% of propolis for the samples heated at temperatures of 30°C, 40°C, 50°C, and 60°C were shown in Figure 2. The influence of the heating temperature on amplitudes (*A*) and integral intensities (*I*) of the EPR lines of the tested propolis samples was presented in Figures 3 and 4, respectively. The high values of linewidths (Δ*B*_pp_: 0.39–1.22 mT) (Figure 2) were caused by strong dipolar interactions between unpaired electrons of free radicals. The linewidths (Δ*B*_pp_) and dipolar interactions were increasing along with rising heating temperature in both tested propolis ointments. The broader EPR lines were observed for the ointment containing 7% of propolis. Dipolar interactions of free radicals were stronger in this ointment. The character of dependence of amplitudes (*A*) and integral intensities (*I*) on the heating temperature was visible in Figures 3 and 4. The temperature dependence of the values of amplitudes (*A*) and integral intensities (*I*) caused a temperature dependence of free radical concentrations (*N*) in the examined ointments.

**Figure 5:**
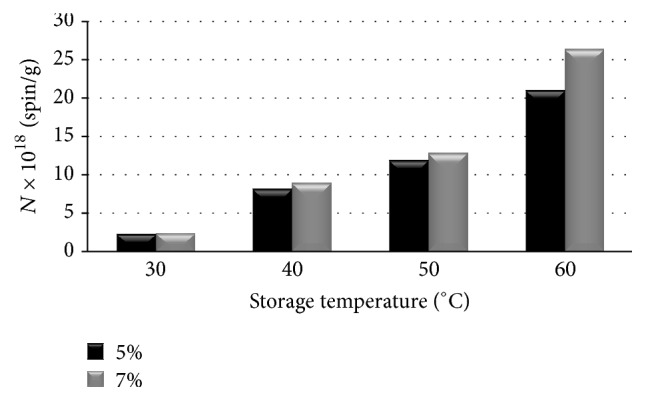
The influence of the heating temperature on free radical concentration (*N*) in the ointments containing 5% and 7% of propolis. The data for the measurements with the microwave power of 2.2 mW.

Free radical concentrations (*N*) in the two examined ointments differing in terms of propolis contents when heated at temperatures of 30°C, 40°C, 50°C, and 60°C were compared in the graph in Figure 5. Free radicals in concentrations of ~10^18^–10^20^ spin/g were detected for the thermally treated propolis samples. Free radical concentrations (*N*) in both ointments (5% and 7% of propolis contents) strongly depended on the heating temperature. The free radical concentrations (*N*) increased with increasing heating temperature of the samples. The differences between free radical concentrations (*N*) in the ointments containing 5% and 7% of propolis treated with temperatures of 30°C, 40°C, and 50°C were not strong. The two tested propolis samples differed in free radical concentrations (*N*) after heating at 60°C. The significantly higher free radical concentrations (*N*) were formed in the ointment containing 7% of propolis compared to the sample containing 5% of propolis. Taking into account the results presented in Figure 5, it was visible that temperatures of 40°C, 50°C, and 60°C were not recommended for storage of propolis ointments. Considering the lowest free radical formation the storage at 30°C was recommended for these samples.

**Figure 6:**
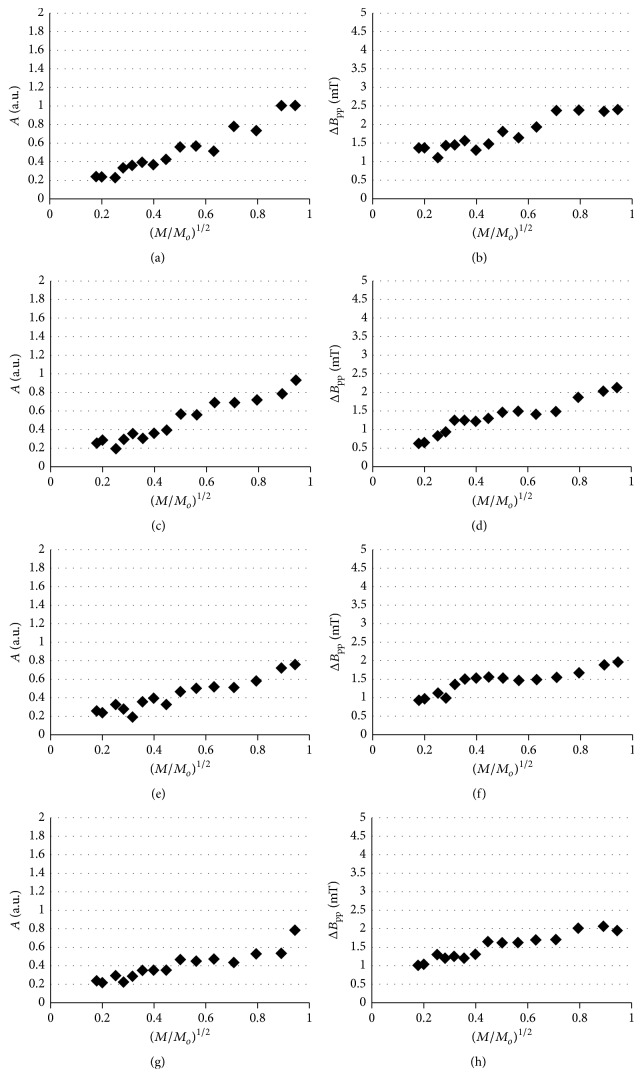
The influence of microwave power (*M*/*M*_*o*_) on (a, c, e, and g) amplitudes (*A*) and (b, d, f, and h) linewidths (Δ*B*_pp_) of the EPR spectra of the ointment containing 5% of propolis thermally treated at temperatures 30°C (a, b), 40°C (c, d), 50°C (e, f), and 60°C (g, h). *M*: microwave power used during the measurement of the EPR spectra; *M*_*o*_: total microwave power produced by klystron (70 mW).

**Figure 7:**
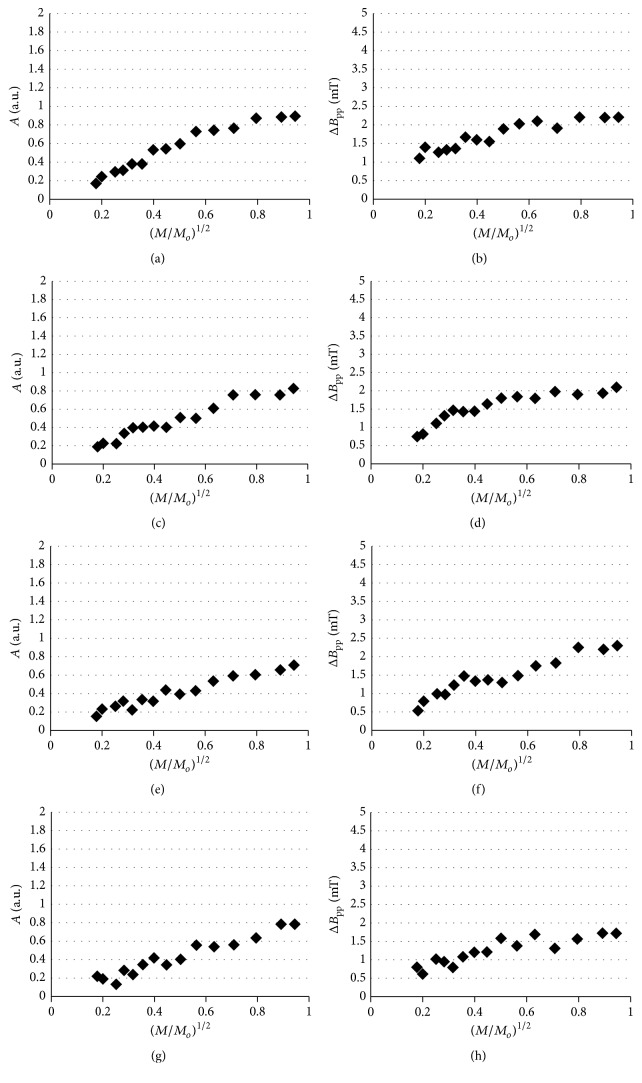
The influence of microwave power (*M*/*M*_*o*_) on (a, c, e, and g) amplitudes (*A*) and (b, d, f, and h) linewidths (Δ*B*_pp_) of the EPR spectra of the ointment containing 7% of propolis thermally treated at temperatures 30°C (a, b), 40°C (c, d), 50°C (e, f), and 60°C (g, h). *M*: microwave power used during the measurement of the EPR spectra; *M*_*o*_: total microwave power produced by klystron (70 mW).

The amplitudes (*A*) and linewidths (Δ*B*_pp_) of the analyzed EPR spectra depended on microwave power (*M*/*M*_*o*_). The changes of amplitudes (*A*) of the EPR lines of the ointment containing 5% of propolis heated at temperatures of 30°C, 40°C, 50°C, and 60°C, with increasing microwave power (*M*/*M*_*o*_), were presented in Figures 6(a), 6(c), 6(e), and 6(g), respectively. The influence of microwave power (*M*/*M*_*o*_) on amplitudes (*A*) of the EPR lines of the ointment containing 7% of propolis heated at temperatures of 30°C, 40°C, 50°C, and 60°C was shown in Figures 7(a), 7(c), 7(e), and 7(g), respectively. The changes of linewidths (Δ*B*_pp_) of the EPR lines of the ointment containing 5% of propolis heated at temperatures of 30°C, 40°C, 50°C, and 60°C, with increasing microwave power (*M*/*M*_*o*_), were presented in Figures 6(b), 6(d), 6(f), and 6(h), respectively. The correlations between linewidths (Δ*B*_pp_) of the EPR lines of the ointment containing 7% of propolis heated at temperatures of 30°C, 40°C, 50°C, and 60°C and microwave power (*M*/*M*_*o*_) were visible in Figures 7(b), 7(d), 7(f), and 7(h), respectively.

The linewidths (Δ*B*_pp_) of the EPR spectra of the ointments containing 5% and 7% of propolis increased along with increasing microwave power (*M*/*M*_*o*_) (Figures 6(b), 6(d), 6(f), 6(h), 7(b), 7(d), 7(f), and 7(h)). This effect was characteristic for homogeneously broadened EPR lines [20, 21]. The amplitudes (*A*) for the ointments containing 5% and 7% of propolis heated at temperatures of 30–60°C increased along with increasing microwave power (*M*/*M*_*o*_) (Figures 6(a), 6(c), 6(e), 6(g), 7(a), 7(c), 7(e), and 7(g)). The absence of the microwave saturation along with the decrease of amplitudes (*A*) at the higher microwave powers confirmed fast spin-lattice relaxation processes in the thermally treated propolis samples. The similar times of spin-lattice relaxation processes in the samples heated at different temperatures indicated that magnetic interactions were unchanged. The spin-lattice relaxation of unpaired electrons was retained at temperatures up to 60°C. However, the increase discussed above of free radical concentrations in higher temperatures (Figure 5) did not allow using them during storage of the examined propolis ointments.

The performed X-band (9.3 GHz) electron paramagnetic resonance examination of the ointments containing propolis brings to light the thermal conditions of their storage. The higher temperatures (40–60°C) which produced high concentration of free radicals in the ointments should be rejected in practice. The safe temperatures of storage of the ointments containing both 5% and 7% of propolis were up to 30°C. This work gives information about free radical properties of the tested propolis samples. The obtained EPR results were important for pharmacy and medicine application of propolis.

The apitherapeutic ointments applied in the present study possess numerous medical applications and properties. It is commonly known that the propolis ointment may be successfully applied in case of female patients with cervical erosions, inflammation of the mucous membrane of the uterus, inflammation of the cervix, and nonspecific inflammation of the vulva and vagina [22].

Many studies have been carried out on the antimicrobial effect of topically applied propolis in case of different skin diseases caused by microorganisms. These include suppurative diseases of the skin caused by staphylococci, such as folliculitis, boils, sweat gland infection, and mixed staphylococcal-streptococcal pathologies, including ecthyma, pyoderma or skin tuberculosis, and various fungal and viral diseases [23]. Earlier studies also demonstrated that topical application of propolis extract turned out to be effective in inhibiting carrageenan-induced rat hind paw edema and, additionally, inhibiting chemotaxis of human polymorphonuclear leukocytes (PMNs), the phenomenon of which may also contribute to the anti-inflammatory effect of ointment containing propolis extract [15]. Furthermore, it was also observed that in the course of phonophoresis the propolis ointment caused the decrease in skin sensitivity, due to the anesthetizing effect on the skin receptors [24].

Propolis ointment management was also reported to stimulate the efficacy of the short stretch bandage compression stocking and the combined venous ulcer treatment which was more effective than Unna's boot compression alone [14]. Pessolato et al. reported in their studies that burn treatment with propolis stimulated the process of tissue regeneration and led to inhibition of local inflammation, which indicates that treatment with the mentioned apitherapeutic was successful in the initiation of the burn healing, and accelerated the biosynthesis of collagen fibres (estimated by morphometry) in all the evaluated periods [25].

Anti-inflammatory, antimicrobial, and regenerating properties of the propolis ointment were also examined in our previous studies. We observed that, according to clinical and histopathological evaluation, propolis ointment accelerates regenerative and reconstructive processes and reduces wound healing time [26]. Furthermore, our previous experimental studies revealed that the apitherapeutic ointment accelerates the burnt tissue repair by stimulation of the glycosaminoglycan accumulation in the wound bed, needed for granulation, tissue growth, and wound closure. Moreover, our previously published studies showed that propolis accelerates chondroitin/dermatan sulfates structure modification responsible for binding growth factors that play a crucial role in the tissue repair process [27, 28]. And, last but not least, we also examined antimicrobial properties of propolis ointment. We observed that propolis topical formulation applied in burn wound treatment displayed higher antimicrobial efficacy than commonly used silver sulfadiazine which was demonstrated by significant reduction in microbial colonization as well as bactericidal properties against the isolated strains [29].

## 4. Conclusions

Free radicals were not found in the tested unheated propolis ointments of diamagnetic character. Electron paramagnetic resonance examination pointed out that all the thermally treated propolis ointments independently of the heating temperature contained free radicals (~10^18^–10^20^ spin/g). Free radicals were responsible for paramagnetism of these samples and for their EPR spectra. The measured EPR spectra were homogeneously broadened lines. The EPR lines of the heated propolis ointments were not saturated up to the microwave power of 70 mW, which indicated fast spin-lattice interactions in the samples. The increase of free radical concentrations in the ointments containing propolis (5% and 7%) with increasing of the heating temperature was observed. Thermal formation of free radicals at temperature 60°C was considerably higher in the ointment containing 7% of propolis than in the sample containing 5% of propolis. Taking into account the values of free radical concentrations, it was concluded that both propolis ointments may be stored at the temperature of 30°C, but they should not be stored at higher temperatures 40°C, 50°C, and 60°C. The usefulness of EPR spectroscopy in optimizing storage temperature for the propolis ointments was confirmed.
